# Long-term *in vitro* 3D hydrogel co-culture model of inflammatory bowel disease

**DOI:** 10.1038/s41598-019-38524-8

**Published:** 2019-02-12

**Authors:** Rasha H. Dosh, Nicola Jordan-Mahy, Christopher Sammon, Christine L. Le Maitre

**Affiliations:** 1 Biomolecular Sciences Research Centre, Sheffield Hallam University, Sheffield, S1 1WB UK; 2 Materials and Engineering Research Institute, Sheffield Hallam University, Sheffield, S1 1WB UK; 3 Department of Anatomy and Histology, Faculty of Medicine, University of Kufa, Kufa, Iraq

## Abstract

The *in vitro* study of the pathogenesis of inflammatory bowel disease (IBD) requires a cell model which closely reflects the characteristics of the *in vivo* intestinal epithelium. This study aimed to investigate the application of L-pNIPAM hydrogel as a scaffold to develop a long-term 3D co-culture model of Caco-2 and HT29-MTX cells under conditions analogous to inflammation, to determine its potential use in studying IBD. Monocultures and co-cultures were layered on L-pNIPAM hydrogel scaffolds and maintained under dynamic culture conditions for up to 12 weeks. Treatments with IL-1β, TNFα, and hypoxia for 1 week were used to create an inflammatory environment. Following prolonged culture, the metabolic activity of Caco-2 monoculture and 90% Caco-2/10% HT29-MTX co-cultures on L-pNIPAM hydrogels were increased, and finger-like structures, similar in appearance to villi were observed. Following treatment with IL-1β, TNFα and hypoxia, ALP and ZO-1 were decreased, MUC2 increased, and MUC5AC remained unchanged. ADAMTS1 was increased in response to hypoxia. Caspase 3 expression was increased in response to TNFα and hypoxic conditions. In conclusion, L-pNIPAM hydrogel supported long-term co-culture within a 3D model. Furthermore, stimulation with factors seen during inflammation recapitulated features seen during IBD.

## Introduction

Inflammatory bowel disease (IBD) such as Crohn’s disease is characterized by increased intestinal permeability due to intestinal mucosal barrier dysfunction, which may be a critical factor in the pathogenesis of IBD^1,2^. Furthermore, increased infiltration of inflammatory cells into the lamina propria and submucosa of the small and large intestines are also observed^3^. Several inflammatory mediators are believed to be associated with the development of IBD. Interleukin-1 beta (IL-1β) and tumor necrosis factor alpha (TNFα) are endogenous proinflammatory cytokines that are increased during inflammation of the mucosa and are involved in the pathogenesis of IBD^4–6^. IL-1β and TNFα are secreted by activated immune cells within the lamina propria during inflammation^7^. Many studies have shown that there is an increased expression of IL-1β and TNFα in intestinal biopsy specimens obtained from patients with IBD^1,4,8,9^. Similarly, hypoxia has been shown to impact on the permeability of intestinal epithelial cells^10^, and O_2_ signaling plays an important role in the response to inflammation^11^. In healthy mucosa of the small intestine, epithelial cells survive in physiological hypoxia, this results from counter-current exchange of blood flow which diminishes oxygen tension along the crypt-villus axis. A steep oxygen gradient exists in the normal intestine where PO_2_ levels in the lamina propria and submucosa are 4–8%, this is further reduced across the epithelial and mucus layer to less than 2% in the intestinal lumen^12–14^. Intestinal epithelial oxygen tension has an important role in intestinal inflammation, which is dysregulated in IBD^11,15^. IBD results in increased hypoxia over the inflamed mucosa due to increased oxygen demands of innate immune cells that are recruited to the site of inflammation^14^.

The normal intestinal epithelium contains a number of different epithelial cell types, derived from adult intestinal stem cells, with a range of metabolic, digestive, and barrier functions^16,17^. The two main cell types lining the intestinal epithelium are absorptive enterocytes and mucus-producing goblet cells^18^. The *in vitro* study of the pathogenesis of IBD requires the use of a cell model demonstrating as closely as possible, the characteristics of the *in vivo* intestinal epithelium. Whilst the use of intestinal organoids would enable the modelling of the intestinal tract with normal cells, these take a prolonged period of time to proliferate and differentiate and exhibit poor long term survival in culture using Matrigel^19^. They have to date, only been utilised to generate small organoids and have failed to form the villi structures and morphology seen in the intestinal tract^20^. Most *in vitro* models have used a single cell type, namely the human intestinal epithelial cell line: Caco-2, which is derived from absorptive cells of human colon adenocarcinoma^21,22^. Caco-2 cells, have been widely used to study absorptive functions and permeability of the intestinal epithelium. However, compared to *in vivo* conditions, these models have many limitations^23,24^. One of these limitations, is that Caco-2 cells form closely linked tight junctions, which resemble those of the colon, rather than the small intestine. This results in poor permeability of the cell membrane. Furthermore, Caco-2 monocultures fail to produce an adherent mucus layer which is essential when recreating an intestinal inflammatory niche^24–27^. Subsequently, this has led to the creation of co-culture models which combine Caco-2 cells with the mucus-producing HT29-MTX cells; which are derived from goblet cells of human colon adenocarcinoma^18,24^.

A number of 2D co-culture systems of the small intestine have been developed^28^. An *in vitro* co-culture model combining Caco-2 cells and goblet-like HT29-H cells were first characterised by Wikman-Larhed and Artursson in 1995^29^. Later Walter *et al*., established an *in vitro* co-culture model using Caco-2 and HT29-MTX cells^17^. *In vitro* monocultures and co-cultures of Caco-2 and HT29-MTX cells have been successfully developed to study the intestinal permeability^27,30,31^. However, when these models are used in monolayer the cells fail to develop the crypt-villus architecture seen in the small intestine. 3D monocultures and co-culture studies using a variety of 3D scaffolds such as: porous silk, collagen, and Poly-lactic-co-glycolic acid (PLGA) have been developed^31–33^. For example, Chen *et al*., co-cultured Caco-2 and HT29-MTX cells on geometrically engineered hollow porous silk scaffolds. Here, the Caco-2 and HT29-MTX cells formed an adherent mucus layer which filled the central lumen of this tube. However, there was a reduction in cell function following a few weeks of culture^31^. Similarly, the culture of Caco-2 cells as a monolayer on 3D fabricated villi-shaped collagen scaffolds was used to study drug absorption. However, the collagen scaffold proved to be a barrier to the diffusion of some drugs and Caco-2 cells were unable to live for prolonged periods as the scaffold degraded^32,34^. In contrast, when Caco-2 and HT29-MTX cells were co-cultured on a 3D PLGA scaffold which was designed to replicate the architecture of the intestinal villi; the co-cultured cells migrated to the tips of the villi and underwent differentiation when stimulated by epidermal growth factor (EGF)^35^. Our research group has previously established 3D *in vitro* culture models of the intestinal epithelium, consisting of monocultures of Caco-2 and HT29-MTX cells layered on L-pNIPAM hydrogel scaffolds studied under dynamic culture conditions^36^. This model supported the 3D culture of these cells generating villus-like structures and promoted differentiation, mimicking the native intestinal epithelium^36^.

A limited number of studies have investigated inflammatory mediators within these culture systems. *In vitro* monolayer culture of Caco-2 cells treated with IL-1β and TNFα resulted in increased permeability of tight junctions^1,37,38^ and the production of inflammatory chemokines, such as IL-8^39^. The effects of proinflammatory cytokines have also been investigated in some co-culture models. Susewind *et al*.^40^ and Leonard *et al*.^41^ developed an inflamed 3D co-culture model of Caco-2 cells cultured with either macrophage like (THP-1) or dendritic cells (MUTZ-3) derived from peripheral blood monocytes, or with human immune cell lines. The macrophages and dendritic cells were embedded in type I collagen layers on a transwell inserts, and Caco-2 cells cultivated on the surface. These co-culture models were subsequently stimulated with IL-1β for 2 days to model the inflamed intestinal mucosa. In these models, the inflamed co-cultures, released higher amounts of IL-8 and increased TNFα expression compared to control Caco-2 monocultures^40,41^. However, unfortunately these studies did not include mucus-producing cells.

Some *in vitro* studies have investigated the effects of hypoxia in simple culture systems, where hypoxia increased the production of IL-1β and TNFα by human peripheral blood mononuclear cells following treatment with endotoxin^42^. Caco-2 cells have also been used to study the intestinal epithelial response to hypoxia^43^. Lima *et al*. studied the effect of *Shigella flexneri* on co-cultured Caco-2 and rat hepatocytes in both normoxia and hypoxia (<1% O_2_), and showed this resulted in apoptosis^44^. Similary, Caco-2 cells cultured on polystyrene and grown under hypoxia at 1% O_2_ showed a significant decrease in brush border membrane expression of β1 integrins, which resulted in decreased *Y. enterocolitica* entry into Caco-2 cells^43^. However, these studies did not consider the effect of pro-inflammatory cytokines and hypoxia conditions on 3D co-culture systems.

Hence, this study aspired to develop a model which represents the cell types seen within the intestine under conditions seen during pathological conditions of IBD. This study aimed to further develop our model system reported recently^36^, which enables the individual culture of Caco-2 and HT29-MTX cells on L-pNIPAM hydrogels, where villus-like structures were seen to spontaneously form^36^. In this paper, the model was developed to investigate a long-term *in vitro* 3D co-culture model to mimic the natural epithelial layers of the native intestine, which was then utilised to investigate conditions representative of inflammation to determine its potential to study disease processes.

## Results

### Metabolic activity of monocultures and co-cultures of Caco-2 and HT29-MTX cells layered on L-pNIPAM hydrogel scaffolds under dynamic culture conditions

**Figure 1:**
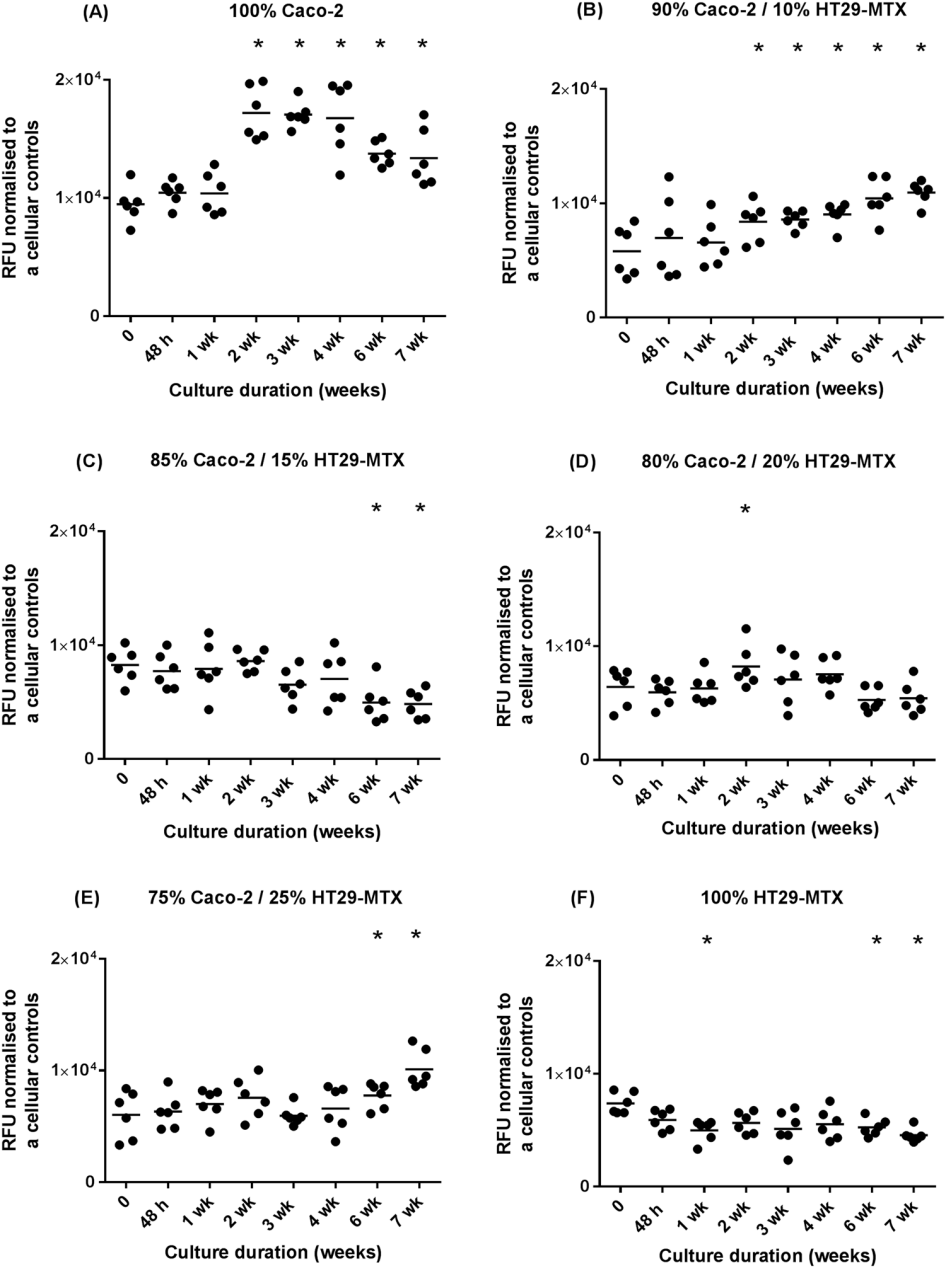
Metabolic activity of monocultures and co-cultures of Caco-2 and HT29-MTX cells at a total cell density of 2 × 10^6^ cells/ml with different percentages. (**A**) Caco-2 cells alone (n = 6); (**B**) 90% Caco-2/10% HT29-MTX (n = 6); (**C**) 85% Caco-2/15% HT29-MTX (n = 6); (**D**) 80% Caco-2/20% HT29-MTX (n = 6); (**E**) 75% Caco-2/25% HT29-MTX (n = 6); (**F**) HT29-MTX cells alone (n = 6) layered on L-pNIPAM hydrogel scaffolds under dynamic culture conditions following 7 weeks. All replicates have been shown with the median value indicated to demonstrate clearly the spread of replicates. *P ≤ 0.05, each time points compared to time 0.

When Caco-2 monocultures were layered on the surface of L-pNIPAM hydrogel scaffolds under dynamic culture, there was a significant increase in metabolic cell activity by week 2 to week 7 (P < 0.0001) (Fig. 1A). In layered co-cultures of 90% Caco-2/10% HT29-MTX, there was a significant increase in metabolic activity from week 2 (P = 0.024), week 3 (P = 0.0184), week 4 (P = 0.0033), week 6 (P < 0.0001) to week 7 (P < 0.0001) (Fig. 1B). In contrast, the metabolic activity of layered co-cultures of 85% Caco-2/15% HT29-MTX significantly decreased from week 6 (P = 0.0184) to week 7 (P = 0.0122) (Fig. 1C). In layered co-cultures of 80% Caco-2/20% HT29-MTX, there was only a significant increase in metabolic activity between day 0 and 2 weeks (P < 0.0001) (Fig. 1D). In layered co-cultures of 75% Caco-2/25% HT29-MTX; there was a significant increase in metabolic activity by week 6 (p = 0.0232) to week 7 (P < 0.0001) (Fig. 1E). When HT29-MTX monocultures were layered alone on L-pNIPAM hydrogel under dynamic culture, there was a significant decrease in metabolic activity from day 0 to 1 week (P = 0.0161), then the metabolic activity remained constant and followed by a significant decrease in metabolic cell activity by week 6 (P = 0.0406) and week 7 (P = 0.0013) (Fig. 1F).

### Morphological and phenotypic assessment of monocultures and co-cultures of Caco-2 and HT29-MTX cells layered on L-pNIPAM hydrogel scaffolds under dynamic culture conditions

**Figure 2:**
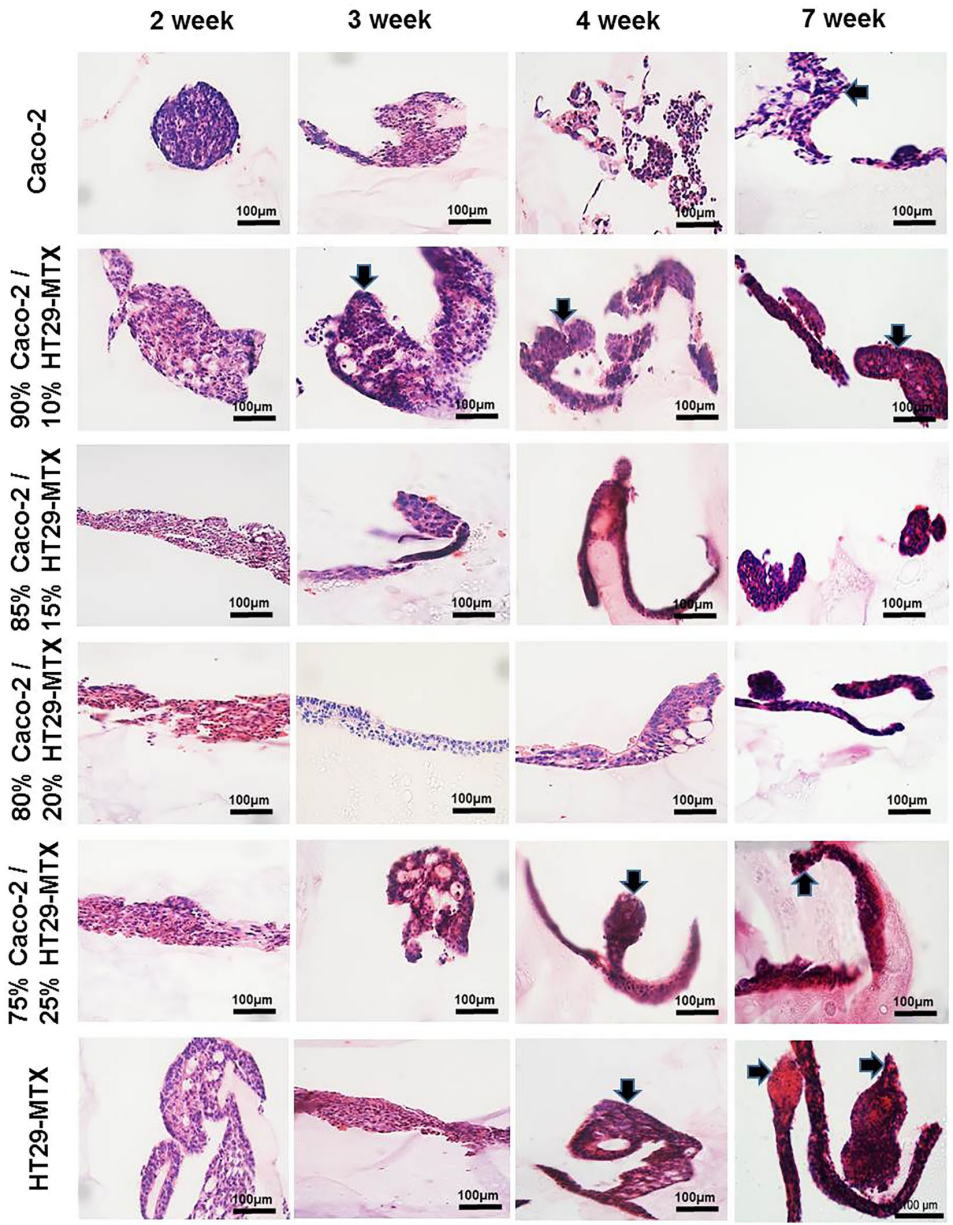
Morphology of monocultures and co-cultures of Caco-2 and HT29-MTX cells at a total cell density of 2 × 10^6^ cells/ml with different percentages layered on L-pNIPAM hydrogel scaffolds under dynamic culture conditions following 7 weeks stained with H&E. The black arrows indicate the finger-like structures. Scale bar = 100 µm.

When Caco-2 monocultures were layered on L-pNIPAM hydrogel scaffold and maintained under dynamic culture conditions, cells showed spheroid structures by 2 weeks and subsequently formed finger-like structures between 3 to 7 weeks in culture (Fig. 2). Layered co-cultures of 90% Caco-2/10% HT29-MTX formed multi-cellular layers over the surface of the hydrogel following 2 weeks. These co-cultured cells were well preserved and following 3 to 7 weeks formed finger-like structures (Fig. 2). However, in layered co-cultures of 85% Caco-2/15% HT29-MTX cells and 80% Caco-2/20% HT29-MTX cells, a multi-cellular layer was formed between 2 and 7 weeks (Fig. 2). In layered co-cultures of 75% Caco-2/25% HT29-MTX, multi-cellular layers were observed following 2 week culture, which went on to form finger-like structures after 4 to 7 weeks of culture (Fig. 2). When HT29-MTX monocultures were layered on L-pNIPAM and maintained under dynamic culture conditions for 7 weeks, multi-cellular layers were observed in cultures between 2 and 3 weeks, which went on to form finger-like structures following 4 to 7 weeks under the same conditions (Fig. 2).

**Figure 3:**
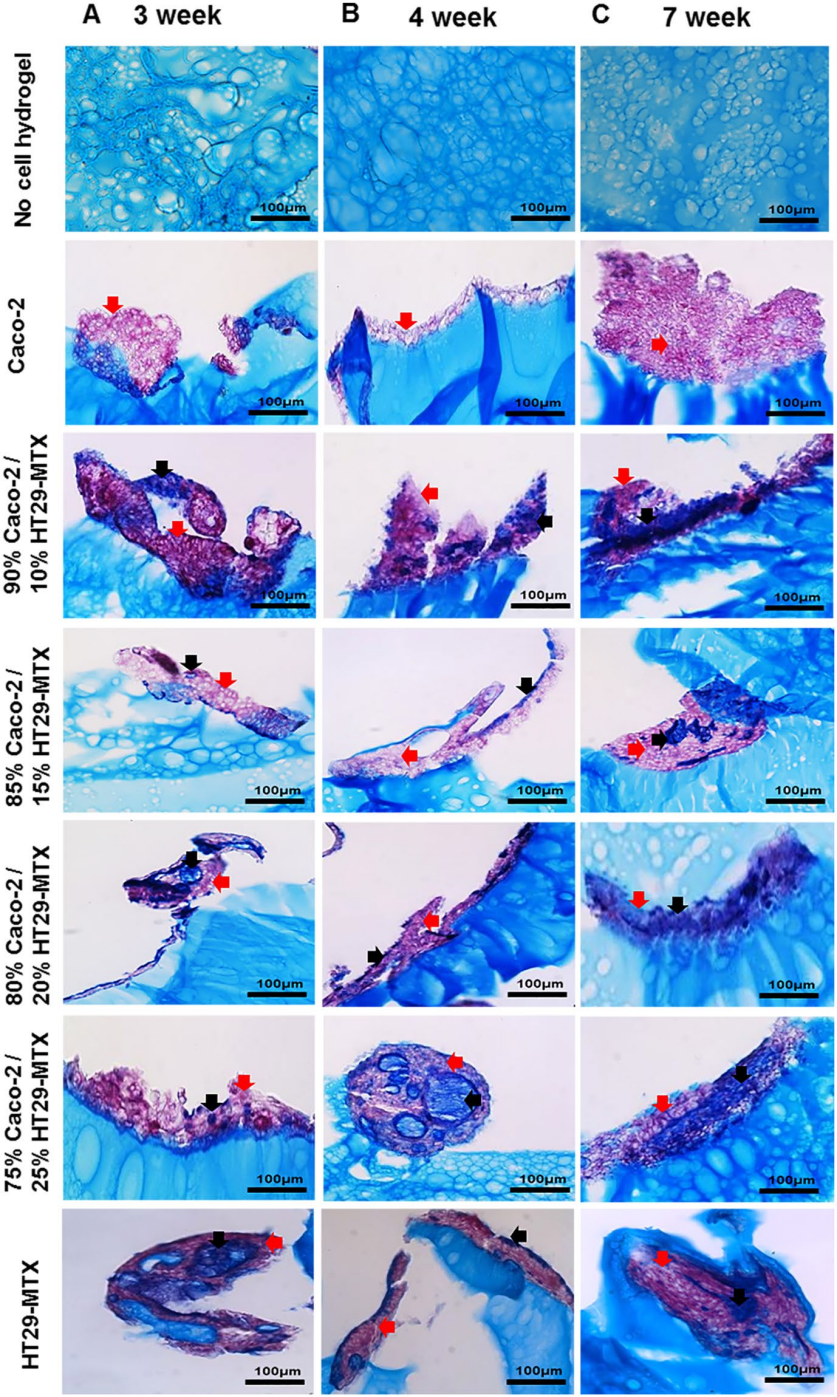
Mucin expression by monocultures and co-cultures of Caco-2 and HT29-MTX cells at different percentages layered on L-pNIPAM hydrogel scaffolds, together with no-cell controls under dynamic culture conditions following (**A**) 3 weeks; (**B**) 4 weeks; and (**C**) 7 weeks stained with AB-PAS, blue: acidic mucin (black arrows); magenta: neutral mucin (red arrows). Scale bar = 100 µm.

To determine potential mucin production, cell cultures following 3, 4, and 7 weeks in culture were stained using Alcian blue and periodic acid Schiffs (AB/PAS). Alcian blue positively stains the hydrogel, due to its negative charge, resulting in what appears to be high background staining (Fig. 3). However, where mucins were produced by cells these were still clearly distinct from the background staining in the hydrogel (Fig. 3). Alcian blue detects acidic mucins (blue) whereas PAS detects neutral mucins (magenta). Caco-2 monocultures were only positive for neutral mucins, whereas both neutral mucins and acidic mucins were observed in all co-cultured models and HT29-MTX monocultures after 3, 4, and 7 weeks in culture. An increase in intensity for acidic mucins was observed in layered co-cultures of 75% Caco-2/25% HT29-MTX and in HT29-MTX monocultures (Fig. 3). From the morphological analysis and metabolic studies 90% Caco-2/10% HT29-MTX, and 75% Caco-2/25% HT29-MTX cell ratios were selected for further analysis. These cultures displayed clear formation of finger-like projections which resembled villi (Figs 2 and 3), and showed evidence of cell proliferation with increased metabolic activity (Fig. 1) in addition to increased cell numbers in H&E stained sections (Fig. 2). Furthermore, these cultures showed clear goblet cell morphology and mucin production within cultures (Fig. 3).

### Scanning electron microscopy of monocultures and co-cultures following 7 weeks

**Figure 4:**
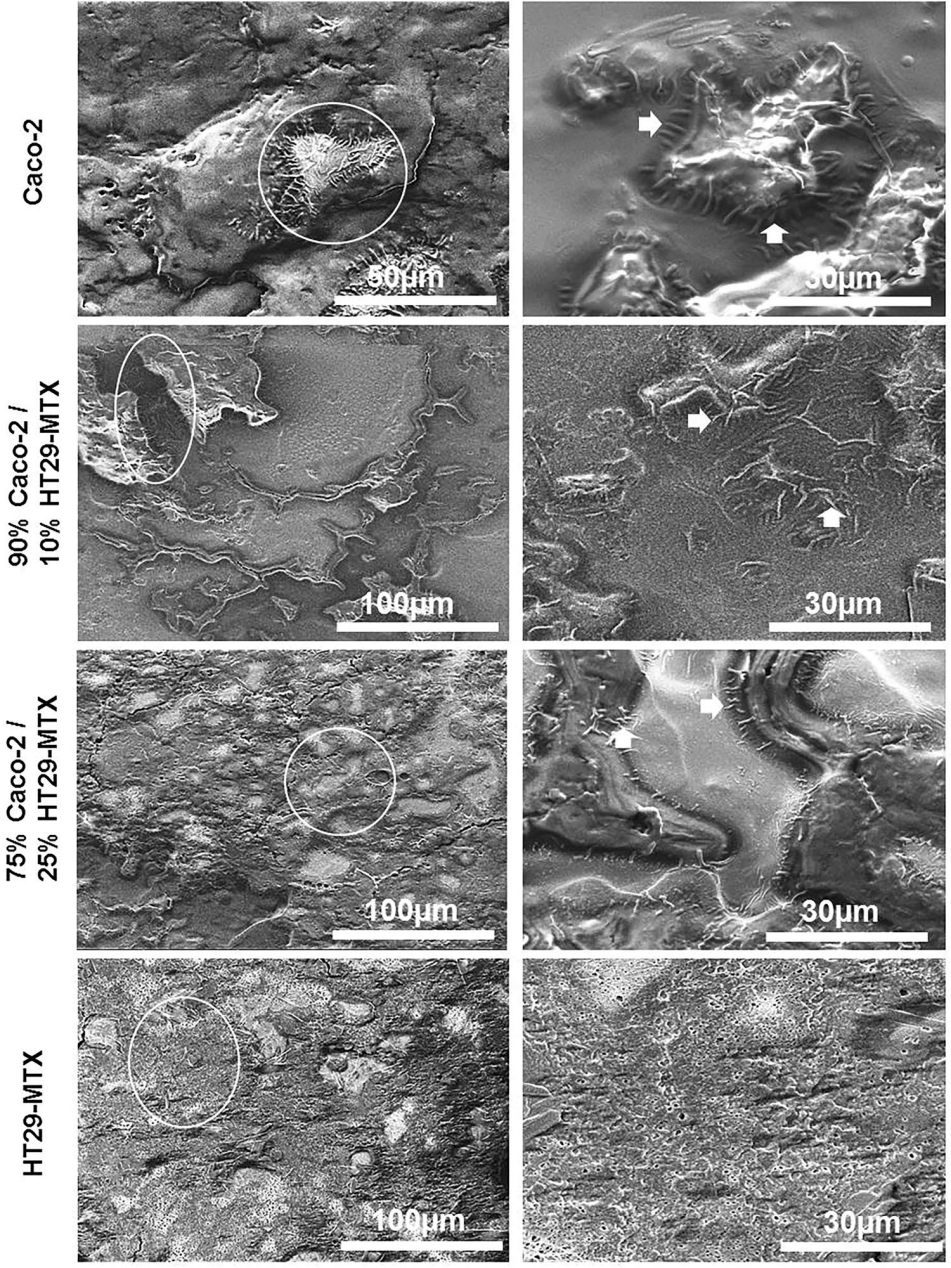
Scanning electron micrographs of monocultures and co-cultures of Caco-2 and HT29-MTX cells at different percentages layered on L-pNIPAM hydrogel scaffolds under dynamic culture conditions following 7 weeks showing microvilli-like structures (white arrows). Scale bar = 30 µm–100 µm.

Examination of the microstructure of monocultures and co-cultures by SEM showed the presence of morphological features which resembled epthieilal microvilli (Fig. 4). These were identified in Caco-2 monocultures, and co-cultures containing: 90% Caco-2/10% HT29-MTX, and 75% Caco-2/25% HT29-MTX following 7 weeks in culture; whilst HT29-MTX monocultures showed budding of the mucus producing goblet-like cells (white arrows) (Fig. 4).

### Treatment of monocultures and co-cultures with pro-inflammatory cytokines or culture under hypoxia

Following 6 weeks of dynamic culture in layers on the L-pNIPAM hydrogel scaffold, monocultures and co-cultures were treated with (a) pro-inflammatory cytokine IL-1β (10 ng/ml) for 1 week under dynamic culture or (b) cultured under hypoxic conditions at 1% O_2_ for 1 week under static culture conditions. Among these cultures, layered co-cultures of 85% Caco-2/15% HT29-MTX, 80% Caco-2/20% HT29-MTX and HT29-MTX monocultures appeared to undergo cell death and cell debris was observed following treatment with 10 ng/ml IL-1β or 1% O_2_ for 1 week (Supplementary Fig. 1). Whilst Caco-2 monocultures, co-cultures of 90% Caco-2/10% HT29-MTX, and 75% Caco-2/25% HT29-MTX cells cultured in layers remained viable for the whole study period (Supplementary Fig. 1). These findings resulted in the selection of 90% Caco-2/10% HT29-MTX and 75% Caco-2/25% for further immunohistochemistry assessment.

**Figure 5:**
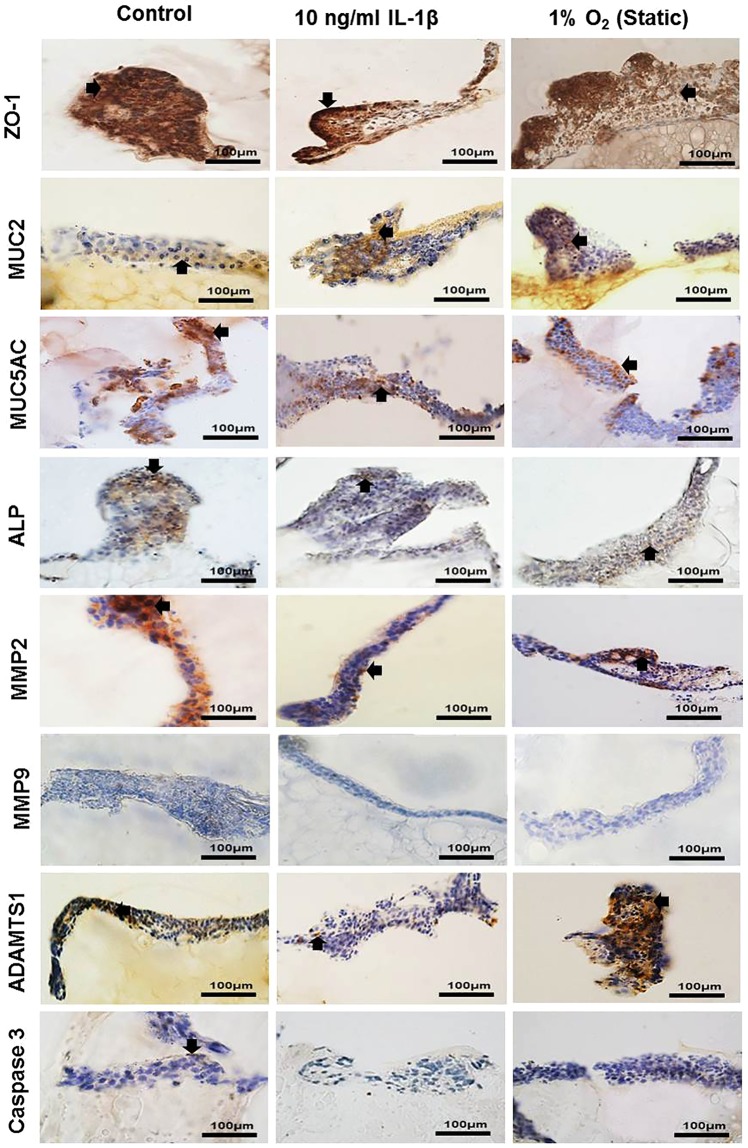
Immunopositivity (brown) of co-culture Caco-2 and HT29-MTX cells at percentages 90% Caco-2/10% HT29-MTX layered on L-pNIPAM hydrogel scaffolds under dynamic culture conditions following 7 weeks as control or for 6 weeks and then treated with 10 ng/ml IL-1β for 1 week under dynamic culture conditions or hypoxic at 1% O_2_ for 1 week under static culture conditions. Cell nuclei were stained with haematoxylin (blue). The black arrows indicate positively stained cells. Scale bar = 100 µm.

To determine features classically associated with IBD, a number of markers were assessed using immunocytochemsitry. In co-cultures containing 90% Caco-2 and 10% HT29-MTX cells, stimulation with IL-1β and 1% O_2_ showed a decrease in Zonulin 1 (ZO-1) expression and increased expression of MUC2 compared to controls, whilst MUC5AC expression remained unchanged in all cultures (Fig. 5). Alkaline phosphatase (ALP) was highly expressed in the control, with less expression in cells treated with IL-1β and 1% O_2_ (Fig. 5). Matrix metalloproteinase 2 (MMP2) expression was decreased in IL-1β treated cells compared to the control and 1% O_2_ cultures (Fig. 5). Immunopositivity for MMP9 was not observed in any system studied here. ADAMTS1 was highly expressed in 1% O_2_ culture compared to the control and IL-1β cultures. Caspase 3 was expressed at low levels in the control, but was not observed in IL-1β and 1% O_2_ treated cultures (Fig. 5).

In co-cultures containing 75% Caco2 and 25% HT29-MTX cells, ZO-1 and MUC2 were highly expressed in the control, with less expression in 1% O_2_ and IL-1β treated cultures (Supplementary Fig. 2). MUC5AC was positively expressed in all cultures in this study. ALP immunopositivity was increased in IL-1β and 1% O_2_ treated cultures compared to control (Supplementary Fig. 2). MMP2 was more highly expressed in the control and 1% O_2_ cultures, than those treated with IL-1β. Immunopositivity for MMP9 was not observed in any of these cultures (Supplementary Fig. 2). ADAMTS1 was highly expressed in 1% O_2_ culture compared to the control and IL-1β treated cultures. Caspase 3 was expressed in 1% O_2_ cultures, but was not observed in control and IL-1β treated cultures (Supplementary Fig. 2).

**Figure 6:**
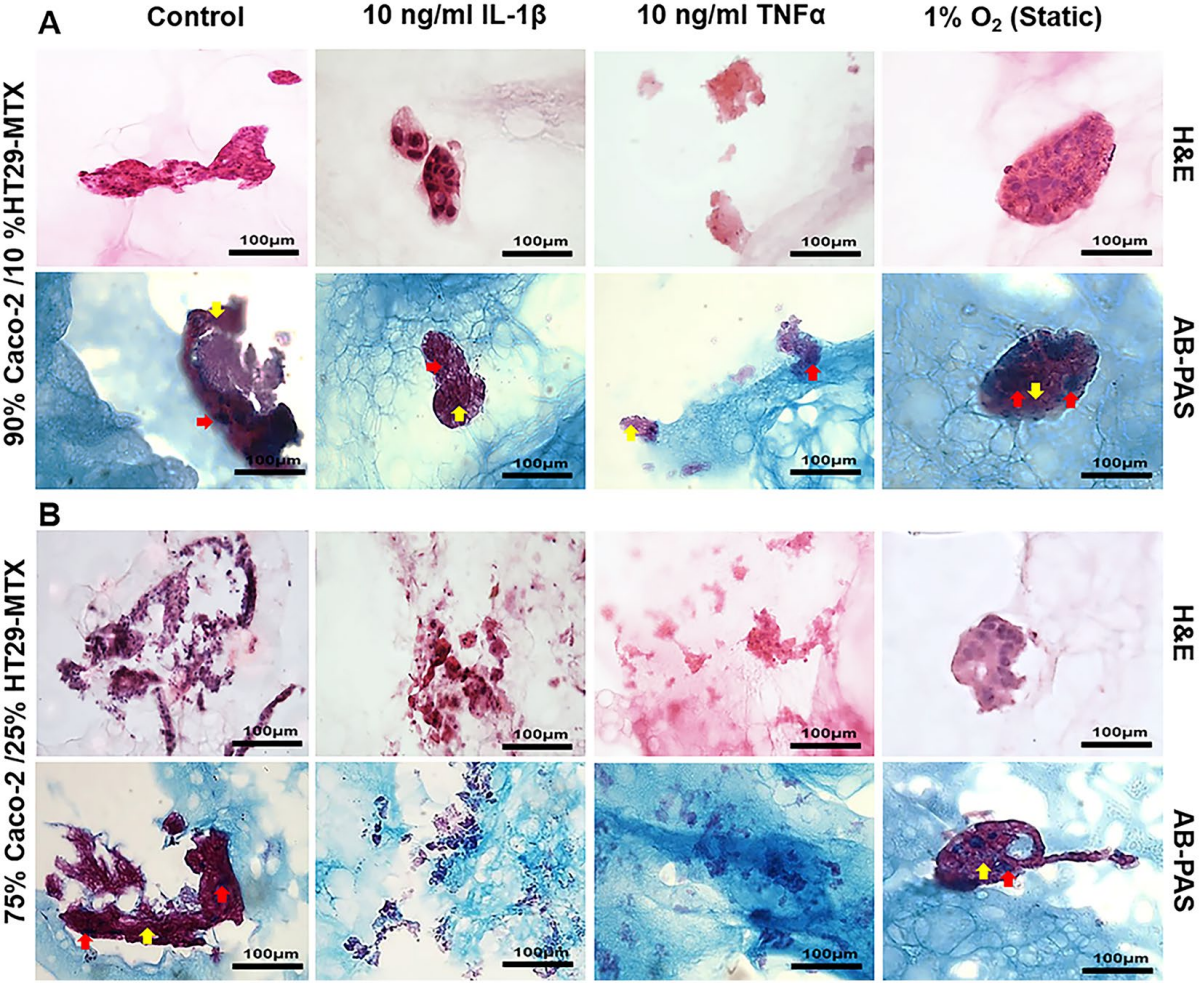
Morphology of long term co-culture of Caco-2 and HT29-MTX cells at percentages: (**A**) 90% Caco-2/10% HT29-MTX; (**B**) 75% Caco-2/25% HT29-MTX layered on L-pNIPAM hydrogel scaffolds under dynamic culture conditions following 12 weeks as control or for 11 weeks and then treated with 10 ng/ml IL-1β or 10 ng/ml TNFα for 1 week under dynamic culture conditions or hypoxic at 1% O_2_ for 1 week under static culture conditions stained with H&E and AB-PAS, blue: acidic mucin (red arrows); magenta: neutral mucin (black arrows). Scale bar = 100 µm

**Figure 7:**
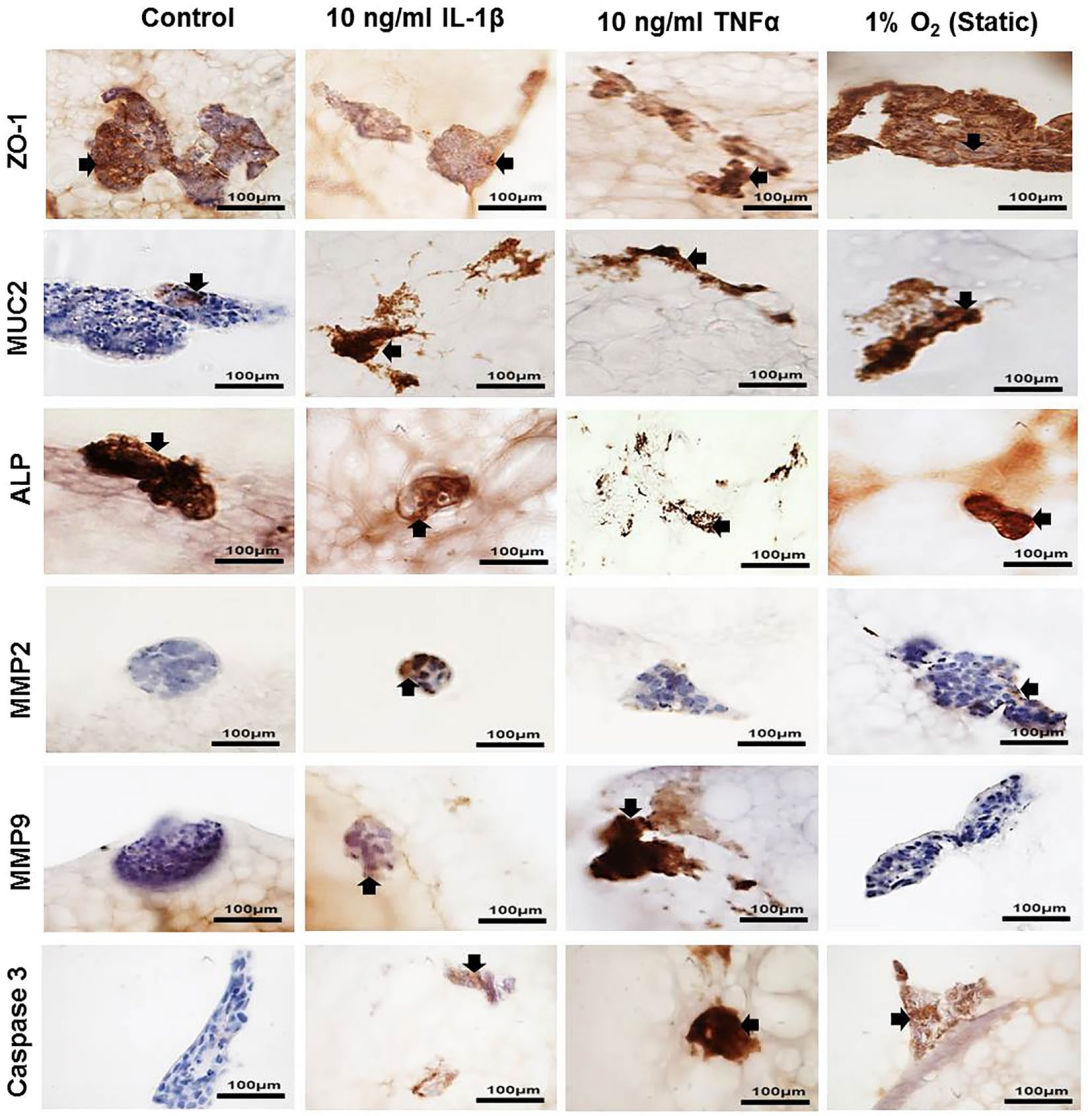
Immunopositivity (brown) of co-culture Caco-2 and HT29-MTX cells at percentages 90% Caco-2/10% HT29-MTX layered on L-pNIPAM hydrogel scaffolds under dynamic culture conditions following 12 weeks as control or for 11 weeks and then treated with 10 ng/ml IL-1β or 10 ng/ml TNFα for 1 week under dynamic culture conditions or hypoxic at 1% O_2_ for 1 week under static culture conditions. Cell nuclei were stained with haematoxylin (blue). The black arrows indicate positively stained cells. Scale bar = 100 µm

To investigate the impact of long-term co-culture on cell behaviour, cell percentages of 90% Caco-2/10% HT29-MTX and 75% Caco-2/25% HT29-MTX cultures were selected for further long term culture. The non-stimulated co-culture of 90% Caco-2/10% HT29-MTX cultured as layers on the surface of L-pNIPAM hydrogels under dynamic culture conditions were well preserved and formed multicellular layers. In contrast, when co-cultured cells were treated with IL-1β, only small cell clusters were seen. Cells in cultures treated with TNFα, showed poor nuclear morphology which is consistent with non-viable cells (Fig. 6A) and this was confirmed by an increase in caspase 3 expression within these cells (Fig. 7). However, when co-cultured cells were maintained at 1% O_2_ for the final week of culture, cells formed multicellular spheroid like structures (Fig. 6A). Neutral and acidic mucins were observed in the control cultures and cultures maintained under 1% O_2_ for the final week of study, whilst lower levels of mucin were seen in cultures treated with IL-1β and TNFα (Fig. 6A). In cultures containing 75% Caco-2/25% HT29-MTX, cells showed evidence of cell death following stimulation with IL-1β and TNFα (Figs 6B and 7). Neutral and acidic mucins were observed in the control and at 1% O_2_ cultures (Fig. 6B). From this morphological assessment, co-cultures containing 90% Caco-2 and 10% HT29-MTX were selected for further investigation of the inflammatory response using immunohistochemistry.

As an indication of inflammation in the 90% Caco-2/10% HT29-MTX co-culture model, ZO-1, MUC2, ALP, MMP2, MMP9 and caspase 3 were investigated in co-cultures stimulated with IL-1β, TNFα, and 1% O_2_ together with un-stimulated controls using immunohistochemistry. ZO-1 was decreased in IL-1β, TNFα and 1% O_2_ treated cultures when compared to the control; suggesting an increase in membrane permeability. IL-1β, TNFα and 1% O_2_ treatment also increased the expression of MUC2 compared to the control (Fig. 7). ALP was highly expressed in the control and 1% O_2_ cultures, compared to those treated with IL-1β and TNFα. MMP2 was expressed in the control and 1% O_2_ cultures but was not observed in IL-1β and TNFα treated cultures (Fig. 7). MMP9 was highly expressed in TNFα treated cultures, with less expression seen in the IL-1β treated cultures, but this was not observed in control and 1% O_2_ cultures. Caspase 3 was highly expressed in TNFα treated cultures compared with IL-1β and 1% O_2_ cultures, but was not observed in controls (Fig. 7).

### Scanning electron microscopy of long-term co-culture following 12 weeks

**Figure 8:**
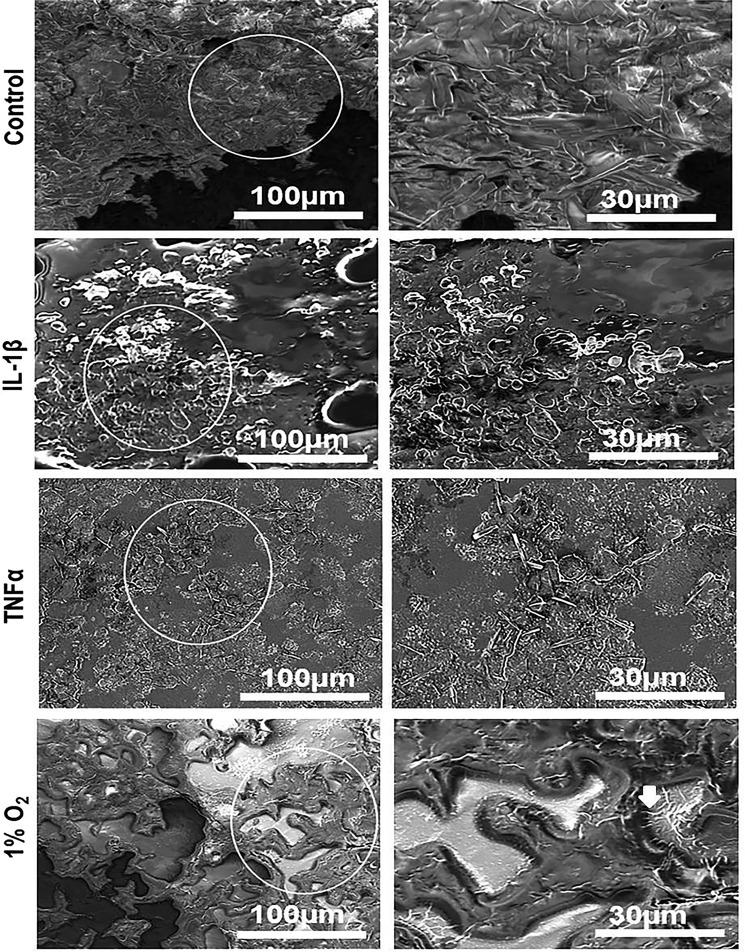
Scanning electron micrographs of long-term co-culture Caco-2 and HT29-MTX cells at percentages 90% Caco-2/10%HT29-MTX cells layered on L-pNIPAM hydrogel scaffolds under dynamic culture conditions following 12 weeks as control or for 11 weeks and then treated with 10 ng/ml IL-1β or 10 ng/ml TNFα for 1 week under dynamic culture conditions or hypoxic at 1% O_2_ for 1 week under static culture conditions. The white arrows indicate the microvilli-like structures. Scale bar = 30 µm, 100 µm.

The SEM observations suggested that the cell morphology and spreading were affected by pro-inflammatory cytokines. When co-cultures containing 90% Caco-2/10% HT29-MTX, and 75% Caco-2/25% HT29-MTX cells were layered on L-pNIPAM hydrogel scaffolds for 12 weeks, the cells were completely spread out with undulation of the cell layer observed in control, whereas co-cultures treated with IL-1β and TNFα were still rounded (Fig. 8 and Supplementary Fig. 3). Hypoxic co-cultures at 1% O_2_ were spread out over the hydrogel with distinct microvilli on their apical surfaces within co-cultures of 90% Caco-2/10% HT29-MTX (white arrows) (Fig. 8). While small rounded cells were observed in 75% Caco-2/25% HT29-MTX co-culture under 1% O_2_ culture conditions (Supplementary Fig. 3).

## Discussion

Co-culture of intestinal epithelial cells: Caco-2 and HT29-MTX have been extensively used as 2D *in vitro* models to study intestinal epithelial barrier functions^18,45,46^. Our previous study demonstrated that L-pNIPAM hydrogel scaffolds supported the 3D layered monocultures of Caco-2 and HT29-MTX cells for 21 days under dynamic culture conditions and maintained cell differentiation and stimulated them to form finger-like architecture, consistent with the structure and size of intestinal villi^36^. Here, the L-pNIPAM hydrogel scaffold was investigated to determine the ability to support long-term co-culture of Caco-2 and HT29-MTX cells under dynamic conditions to develop an *in vitro* intestinal epithelium model. This model was then treated with proinflammatory cytokines IL-1β, TNFα, or hypoxic conditions at 1% O_2_ to mimic the environment observed during inflammatory bowel disease^15,47^.

To characterise the 3D co-culture model *in vitro*, the metabolic activity, and phenotype of these cultures were assessed. The metabolic activity of Caco-2 monoculture or 90% Caco-2/10% HT29-MTX co-cultures was increased when layered on L-pNIPAM hydrogel scaffolds under dynamic culture. Cells formed a multi-cellular layer which became undulated and gave rise to finger like projections which resembled villi. Importantly, this was not observed when co-cultures contained more than 10% HT29-MTX cells. This contradicts previous work in which HT29-MTX cells were found to have a faster proliferation rate than Caco-2 cells grown in monolayers^48^. The mono- and co-culture of Caco-2 and HT29-MTX cells differentiated following 3 weeks. This was evident by the expression of ALP and the presence of microvilli-like structures of Caco-2 cells (observed via SEM), plus the expression of MUC2 by HT29-MTX cells. However, dynamic culture conditions using an orbital shaker resulted in the formation of non-aligned microvilli. Thus, the utilisation of a perfusion bioreactor system may be useful to enable the development of one-directional microvilli.

For long-term co-culture experiments, the optimal seeding percentages were 90% Caco-2/10% HT29-MTX cells and 75% Caco-2/25% HT29-MTX cells. Both HT29-MTX cells and Caco-2 cells were shown to produce mucus. This agreed with our previous observations^36^ and those of Mahler *et al*. who cultured 90% Caco-2/10% HT29-MTX and 75% Caco-2/25% HT29-MTX cell cultures in monolayer^49^.

Inflammatory bowel diseases such as Crohn’s disease and ulcerative colitis are represented by chronic inflammatory diseases that can affect the intestine by increasing intestinal paracellular permeability resulting in alterations of function and expression of tight junction proteins such as ZO-1^50^. Several inflammatory cytokines play a crucial role in the development of IBD including IL-1β and TNFα. These important inflammatory cytokines have been linked to the dysfunctional intestinal epithelium which leads to permeability defects, which as a key symptom of IBD^51,52^. Thus, *in vitro* cell monolayers have often been utilised to investigate their effects on epithelial permeability using trans-epithelial electrical resistance (TEER), where Caco-2 or HT29 cells on semi-permeable filters form luminal and basolateral compartments and the electrical resistance is measured between compartments^53^. IL-1β and TNFα expression in patients with Crohn’s disease has been shown to lead to increased intestinal permeability^54,55^. However, few studies have investigated the role of IL-1β and TNFα in intestinal permeability within 3D culture systems. In this study, as a proxy measure of intestinal permeability, expression of ZO-1 was utilised due to its role in maintaining barrier function^56^. This study showed IL-1β and TNFα reduced ZO-1, which would lead to an increase in paracellular permeability of co-culture following 7 and 12 weeks compared to control and to untreated Caco-2 monoculture following 3 weeks, in our previous study^36^. This observation is consistent with previous studies by Wang *et al*.^57^, and Al-sadi *et al*.^37^, where IL-1β and TNFα increased Caco-2 tight junction permeability by inducing increases in Myosin L Chain Kinase (MLCK) expression and activity^37,57^.

The adherent mucus layer provides protective function for intestinal mucosa against physical and chemical injury, assisting in the clearance of pathogens, and plays an important role in maintaining mucosal integrity^58,59^. Alteration in mucin production is detected in patients with IBD^60^. In our co-culture models, MUC2 was produced following 7 and 12 weeks in co-cultures with 90% Caco-2/10% HT29-MTX. Expression of MUC2 was increased when these cultures were treated with IL-1β and TNFα compared to control, levels of MUC2 were also seen to be greater in these samples than untreated single cultures of HT29-MTX cells following 3 weeks in our previous study^36^. This agrees with previous studies which have shown IL-1 and TNFα stimulate mucin secretion via IL-1R1 on the basolateral surface of cultured HT29-C1.6E cells and via NF-KappaB pathway in HM3 colon cancer cells^61,62^.

Intestinal ALP is a brush border membrane protein which is expressed by enterocytes and is used as a marker for crypt-villus differentiation^63^. Our results showed the expression of ALP was increased with the increase in the percentage of Caco-2 cells in co-cultures, with monocultures of Caco-2 cells expressing high levels of ALP^36^. The 90% Caco-2/10% HT29-MTX co-cultured cells produced the highest level of ALP activity and hence suggests differentiation of the Caco-2 cells to enterocyte-like cells. As a consequence, these co-culture percentages were used in our further study of pro-inflammatory conditions. Nollevaux *et al*. developed a co-culture model compromising 75% Caco-2/25% HT29-MTX grown in serum-free medium^25^. This model produced monolayers which expressed ALP, suggesting that differences in culture conditions, as well as the percentage of each cell line, may affect cell differentiation and the expression of ALP. Subsequently, here all investigations of inflammation in our co-culture model were performed in 90% Caco-2/10% HT29MTX and 75% Caco-2/25% HT29MTX co-cultures.

An increased expression of MMPs is associated with intestinal inflammation and Crohn’s disease. Here, we investigated the expression of MMP2 and MMP9 as these are biomarkers for inflammation^64^. MMP9 activity has previously been shown to increase following TNFα overproduction by intestinal epithelial cells in TNF^ΔARE/+^ murine model, whereas MMP2 activity was not altered in this model^65^. Studies have also shown that high levels of MMP9 are acompanied by low levels of MMP2 in inflamed mucosa^66^. IL-1β and TNFα have been shown to induce synthesis of MMP2 and MMP9 in inflammatory cells and epithelial cells^67^. Gan *et al*. demonstrated that MMP9 was highly expressed in Caco-2 cells stimulated with IL-1β and TNFα^68^. In this study, the observed increased expression of MMP2 in control cultures could be due to the fact that Caco-2 and HT29-MTX cells are tumourgenic, and expression and activity of MMPs are upregulated in many cancerous cells^69^.

Here, we also investigated the expression of ADAMTS 1, which plays a critical role in inflammation^70^. In normal tissues, ADAMTS1 is not highly expressed, but increases during inflammation^71^. In our model, ADAMTS1 was highly expressed in control cells compared to co-cultures treated with IL-1β. This upregulation could be due to the fact that Caco-2 and HT29-MTX cells are cancer cell lines and the proteolytic activity of ADAMTS1 has been related to local tissue invasion in cancer^72^. These findings are in agreement with those of Demircan *et al*.^73^ and Kalinski *et al*.^74^, who reported a decrease in ADAMTS1 expression in a chondrosarcoma cell line (OUMS-27 and C3842) stimulated with IL-1β. However, Filou *et al*. stated that RNA levels of ADAMTS1 were upregulated in the muscular tissue of healthy colons, while downregulated in colon cancer^72^. Additionally, in this study, western blot analysis showed that ADAMTS1 protein was produced by both Caco-2 and HT-29 cells^72^.

The normal intestinal epithelium has been shown to be in a physiologic state of hypoxia^12^. Intestinal epithelial cells respond to hypoxia, through the transcription factor hypoxia-induced factor (HIF-1α and HIF-2α), which are essential in maintaining intestinal homeostasis. Intestinal epithelial cells express high amounts of HIF-1α and HIF-2α in Crohn’s disease^11,13,15^. In the current study, we examined the influence of hypoxia on cell behaviour in monocultures and co-cultures. Following 7 weeks, histological assessment of Caco-2 monocultures and co-cultures of 90% Caco-2/10% HT29-MTX showed that 1% O_2_ hypoxia for 1 week had no effect. Only in the 100% HT29-MTX monoculture was there an increase in cell debris and cell death. Under hypoxic conditions, decreased expression of ZO-1 in 90% Caco-2/10% HT29-MTX and 75% Caco-2/25% HT29-MTX co-cultures following 7 weeks and 12 weeks were observed. These findings are in agreement with previous work, where hypoxia decreased the expression and organization of tight junction protein ZO-1^75^; and this was attributed to expression of MLCK^75^. In contrast, we observed an increase in expression of MUC2 in 90% Caco-2/10% HT29-MTX co-culture following 7 weeks and 12 weeks culture under hypoxia. Similarly, a recent study showed increased MUC2 expression in response to hypoxia in LS174T colorectal cancer cells^76^. Importantly, hypoxia is an important factor that induces cancer metastasis^77,78^, following 7 weeks, hypoxia increased expression of MMP2 and ADAMTS1. This has also been observed in breast carcinoma cells where hypoxia-induced invasion by increased expression of MMP2^78^, in contrast hypoxia had no influence on ADAMTS1 transcription in the chondrosarcomas cell line (C3842)^74^. In this study under hypoxic conditions, apoptotic cells were less prevalent in 90% Caco-2/10% HT29-MTX than the 75% Caco-2/25% HT29-MTX co-culture. Caco-2 cells consume less oxygen under hypoxia and thus may be better adapted to hypoxic conditions^44^.

The successful application of an *in vitro* model depends on how closely this model mimics the characteristics of the *in vivo* intestinal epithelium; we developed an intestinal epithelial model that could mimic healthy and diseased conditions. The L-pNIPAM hydrogel scaffold and dynamic culture condition makes long-term co-culture possible and allows the investigation of pro-inflammatory cytokine effect on intestinal cell behaviour. However, the ability of L-pNIPAM hydrogel as a scaffold for more physiologically relevant intestinal cells such as intestinal organoids derived from intestinal stem cells rather than the cancerous cells used in this study would be important to investigate.

## Methods

### Synthesis of L-pNIPAM hydrogel scaffolds

Synthesis of L-pNIPAM hydrogel was performed as described previously^36^.

### Cell lines and culture conditions

The human intestinal epithelial cell lines: Caco-2, (passage 18–29), and HT29-MTX cells, (passage 25–32), were obtained from the American Type Culture Collection (ATCC). Both cells were grown in DMEM (Life Technologies, Paisley, UK) supplemented with 20% (v/v) foetal bovine serum (FBS) (Life Technologies, Paisley, UK) for Caco-2 cells and 10% (v/v) FBS for HT29-MTX cells, 100 U/M penicillin, 100 µg/ml streptomycin (Life Technologies, Paisley, UK), 250 ng/ml amphotericin (Sigma, Poole, UK), 2 mM glutamine (Life Technologies, Paisley, UK). Cells were maintained at 37 °C in a humidified incubator with 5% CO_2_ atmosphere.

### Monocultures and co-cultures of Caco-2 and HT29-MTX layered on L-pNIPAM hydrogel scaffolds

Six ratios of cultured cells were investigated: 100% Caco-2 monocultures, 100% HT29-MTX monocultures and co-cultures of these cells in various initial seeding percentages (90% Caco-2/10% HT29-MTX, 85% Caco-2/15% HT29-MTX, 80% Caco-2/20% HT29-MTX, 75% Caco-2/25% HT29-MTX) in order to compare the cell line models and to determine the optimal cell density required to resemble the cell diversity of the intestinal epithelium. The higher and lower percentages mimic those seen *in vivo* in the small (90%/10%) and large intestine (75%/25%)^49,79^. To prepare layered cultures, 300 µL of liquid L-pNIPAM at 38–39 °C was added to each well of the 48 well plates and 100 µL was added to each well of 96 well plates. Gelation of the L-pNIPAM was induced by cooling to below 32 °C. Following gelation 300 µL or 100 µL of 2 × 10^6^ total cells/ml (for each of the 6 cell culture suspensions) in complete media were applied to the surface of hydrogel scaffolds in 48 and 96 well plates respectively. Following a 30 minute cell attachment period a further 200 µL or 150 µL complete media (Caco-2 culture media) was added to each well. All constructs were incubated at 37 °C and 5% CO_2_ under dynamic conditions using an orbital shaker at 30 rpm, with media replaced every 48 h. Metabolic activity assessments utilized 96 well plates and were determined using Alamar Blue assay following 0 h, 48 h, and weekly between 1–7 weeks. For histological assessment 48 well plate cultures were utilised. Cultures were maintained for an initial 6 weeks in standard culture conditions, following initial model development, cultures were divided into 3 groups as: (1) control; (2) treated with 10 ng/ml recombinant human IL-1β (Peprotech, London, UK), (concentrations were selected from prior studies^38,40^) and (3) cultured under hypoxic conditions in an oxygen controlled glove box (Coy Lab products, York, UK) at 1% O_2_ (under static culture conditions) for a further week prior to harvest.

### Long-term co-cultures

Cell percentages of 90% Caco-2/10% HT29-MTX, and 75% Caco-2/25% HT29-MTX cultures were selected for long-term co-culture. These cultures were also treated with pro-inflammatory cytokines and hypoxia conditions to mimic inflammatory conditions. Cultures were either maintained for 12 weeks in standard media and dynamic culture as a control. Alternatively, following 11 weeks in standard culture, co-cultures were treated with pro-inflammatory cytokines: 10 ng/ml recombinant human IL-1β (Peprotech, London, UK); or 10 ng/ml recombinant human TNFα (Peprotech, London, UK) (concentrations were selected from prior studies^2,80^), or 1% O_2_ in an oxygen controlled glove box at 1% O_2_ (under static culture conditions) for a further week in culture prior to harvest.

### Alamar blue assessment of metabolic activity

The metabolic activity of monocultures and co-cultures layered on L-pNIPAM hydrogel scaffolds under dynamic culture conditions were assessed using Alamar blue assay (Life Technologies, Paisley, UK) in complete media following 0–7 weeks of culture using the manufacturer’s protocol. The fluorescence intensity was measured using an excitation wavelength of 590 nm via a fluorescence microplate reader (CLARIOstar®, BMG LABTECH). Relative fluorescence units (RFU) were recorded for cellular hydrogel scaffolds and normalized to RFU of acellular hydrogel scaffolds as an indication of total live cells.

### Histological analysis

Monocultures and different percentages of co-cultures in 48 well plates were utilised for histological analysis. Triplicate samples for each culture condition and time point (2, 3, 4, 7, and 12 weeks) were processed to paraffin embedded sections as previously described^36^. Haematoxylin and Eosin (H&E), Alcian blue/periodic acid Schiff (AB-PAS) were performed following standard protocols.

The slides were examined with an Olympus BX51 microscope and images captured by digital camera and Olympus cell Sens Standard software (Media Cybernetics, Buckinghamshire, UK).

### Immunohistochemical assessment

Due to the L-pNIPAM hydrogel scaffold being non-biodegradable^81^, it was not possible to extract RNA/protein and thus quantifying gene/protein expression was not possible. Consequently qualitative changes relating to the presence or absence of immunohistochemical markers are reported. Immunohistochemistry was performed on co-cultures containing 90% Caco-2/10% HT29-MTX, and 75% Caco-2/25% HT29-MTX following 7 weeks and co-cultures containing 90% Caco-2/10% HT29-MTX following 12 weeks under the various culture conditions. Immunohistochemistry for Zonulin 1 (ZO-1) was used as a marker for tight junction formation. MUC2, together with MUC5AC, expression was used as a marker for HT29-MTX cellular differentiation. Alkaline phosphatase (ALP) was used as a marker for Caco-2 cellular differentiation. Matrix degrading enzymes (MMP2, MMP9 and ADAMTS1); alongside caspase 3 activity were also determined. Immunohistochemistry was performed as described previously^36^ using anti ZO-1 antibody (1:50, rabbit polyclonal) (ab217334 Abcam, Cambridge, UK); anti MUC2 antibody (1:100 rabbit polyclonal) (sc 59859 Santa Cruz, Heidelberg, Germany); anti MUC5AC antibody (ab3649 1:200, mouse monoclonal) (Abcam, Cambridge, UK); anti alkaline phosphatase antibody (1:200 rabbit polyclonal) (ab108337 Abcam, Cambridge, UK); anti MMP2 antibody (1:800 rabbit polyclonal) (ab37150 Abcam, Cambridge, UK); anti MMP9 antibody (1:25 rabbit polyclonal) (ab38898 Abcam, Cambridge, UK); anti ADAMTS1 antibody (1:200 rabbit polyclonal) (ab39194 Abcam, Cambridge, UK); anti Caspase 3 antibody (1:400 rabbit polyclonal) (ab4051 Abcam, Cambridge, UK). Negative controls in which rabbit and mouse IgGs (Abcam, Cambridge, UK) replaced the primary antibody at an equal IgG concentration were used. The slides were examined with an Olympus BX51 microscope and images captured by digital camera and Olympus cell Sens Standard software (Media Cybernetics, Buckinghamshire, UK).

### Scanning electron microscopy for monocultures and co-cultures

Following 7 and 12 weeks in culture, constructs cultured with 90% Caco-2/10% HT29-MTX, and 75% Caco-2/25% HT29-MTX were processed for scanning electron microscopy (SEM) as described previously^36^. The cells were examined using a FEI NOVA nano-200 scanning electron microscope.

### Statistical analysis

All metabolic activity assessments were performed at least 6 times. Normality of data was tested using a Skewness, Kurtosis, Royston Chi-sq, Shapiro Wilk W and Shapiro-Francia W tests, together with a q-q plot. From this analysis, it was demonstrated that the data sets were from mixed populations with some populations displaying potential normal distribution, but others were shown to be not normally distributed, as such non-parametric tests have been performed for all data. Therefore, statistical comparisons were performed by Kruskal-Wallis with a pairwise comparison (Conover-Inman). Each time point was compared to time 0 of monoculture and co-culture for 7 weeks, for the Alamar blue assay with statistical significance accepted at P ≤ 0.05. All replicates have been displayed with the median value indicated to clearly show the spread of replicates.

## Supplementary information

Supplementary figures

## Data Availability

All data generated or analyzed during this study are included in this published article (and its supplementary information files), raw data are available on request from the corresponding author.
