# Supplementary material for: Long-term in vitro 3D hydrogel co-culture model of inflammatory bowel disease
Source: Sci Rep. 2019 Feb 12;9:1812. doi: 10.1038/s41598-019-38524-8 (PMC6372635; doi:10.1038/s41598-019-38524-8)
Supplement: Supplementary file 1 — Supplementary figures [file 41598_2019_38524_MOESM1_ESM.pdf]

Long-term in vitro 3D hydrogel co-culture model of inflammatory bowel disease.

Rasha H. Dosh1, 3, Nicola Jordan- Mahy1,

Christopher Sammon2, Christine L. Le Maitre1,*

Supplementary Figures

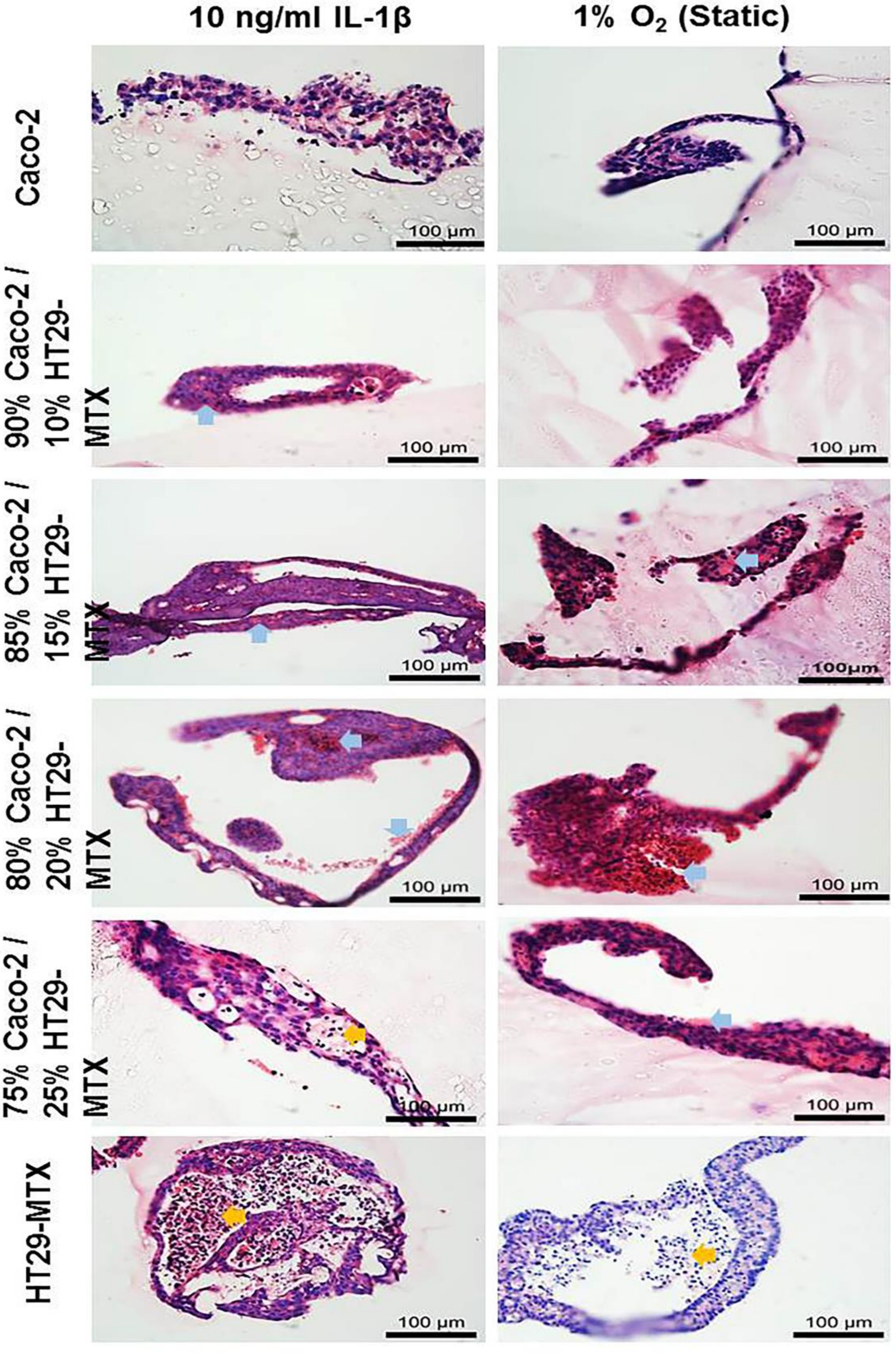

Supplementary Fig. 1. Morphology of monocultures and co-cultures of Caco-2 and HT29-MTX cells at different percentages layered on L-pNIPAM hydrogel scaffolds under dynamic culture conditions for 6 weeks and then treated with 10ng/ml IL-1β for 1 week under dynamic culture conditions or hypoxic at 1% O_2 for 1 week under static culture conditions stained with H&E, blue arrows indicate debris cells, yellow arrows indicate dead cells.
Scale bar = 100μm

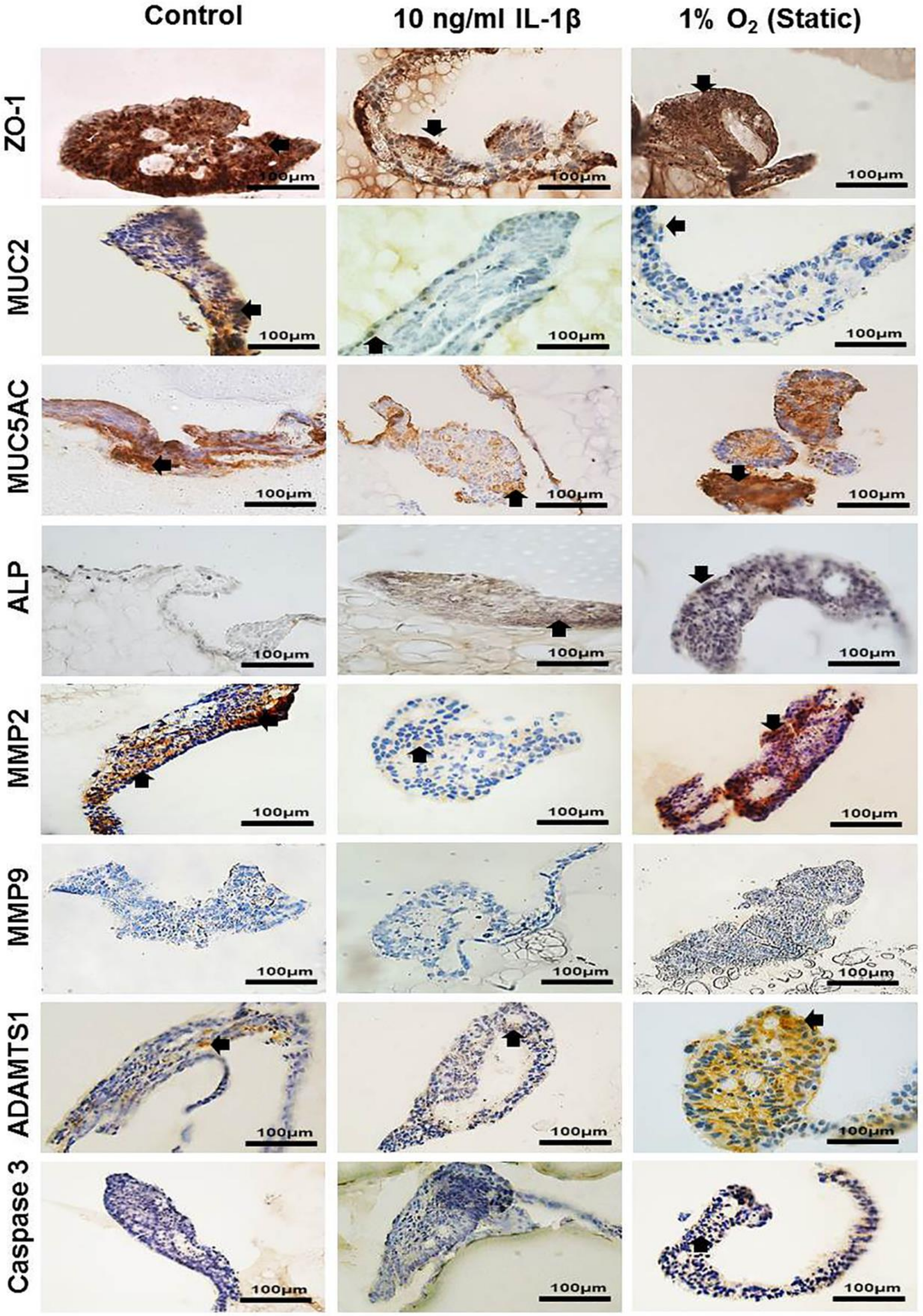

Supplementary Fig. 2. Immunopositivity (brown) of co-culture Caco-2 and HT29-MTX cells at percentages of 75% Caco-2 / 25% HT29-MTX layered on L-pNIPAM hydrogel scaffolds following 7 weeks as control or for 6 weeks and then treated with 10ng/ml IL-1β for 1 week under dynamic culture conditions or hypoxic at 1% O_2 for 1 week under static culture conditions. Cell nuclei were stained with haematoxylin (blue). black arrows showing positively stained cells. *Scale bar = 100μm*

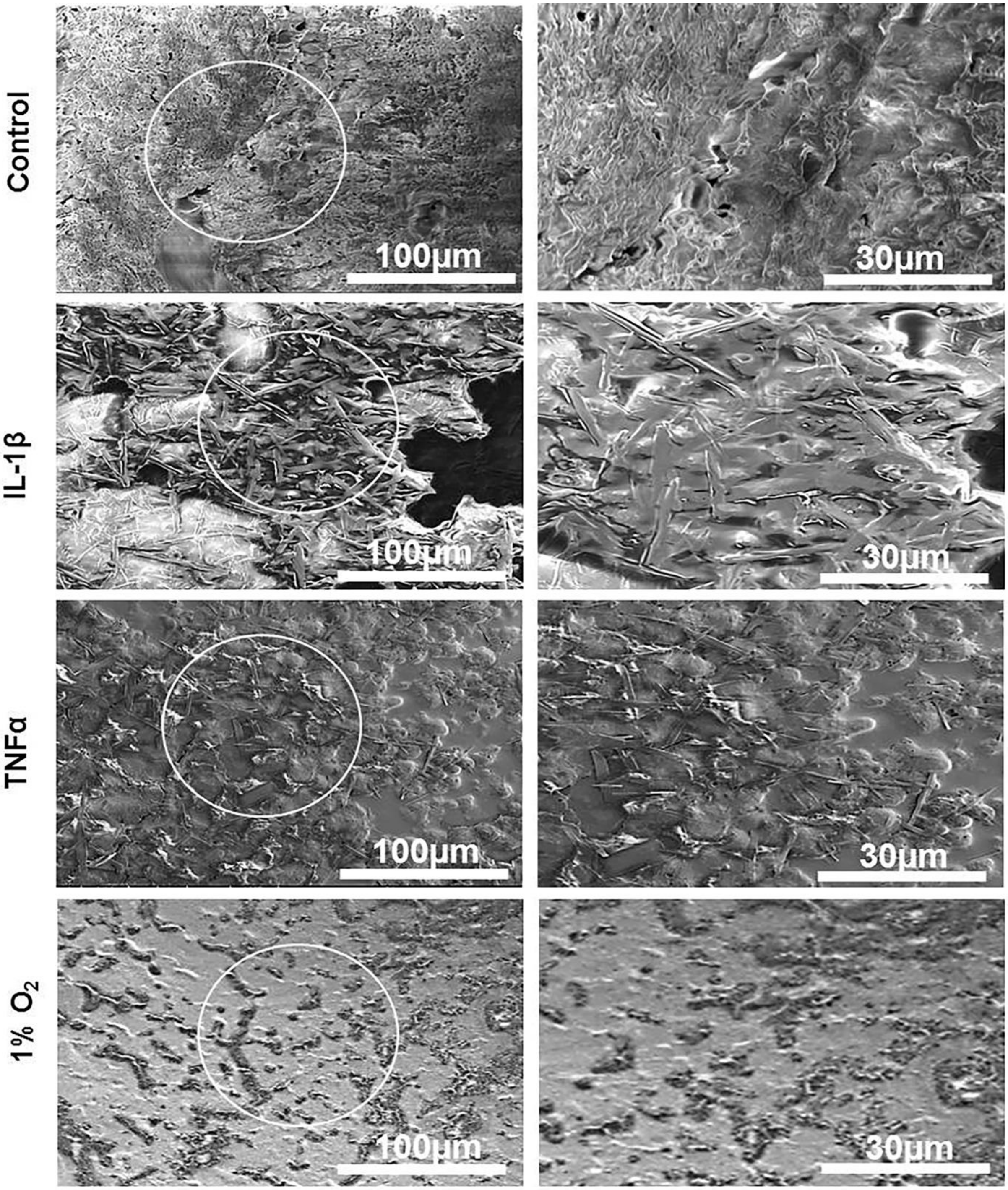

Supplementary Fig. 3. Scanning electron micrographs of long term co-culture Caco-2 and HT29-MTX cells at percentages 75% Caco-2 / 25% HT29-MTX cells layered on L-pNIPAM hydrogel scaffolds under dynamic culture conditions following 12 weeks as control or for 11 weeks and then treated with 10ng/ml IL-1β or 10ng/ml TNFα for 1 week under dynamic culture conditions or hypoxic at 1% O_2 for 1 week under static culture conditions. *Scale bar =100µm, 30µm.*
